# Evaluation of Mortality Rate Disparities for Cardiac Arrest Between Urban and Rural Cohorts in the United States Using the Centers for Disease Control and Prevention Wide-Ranging Online Data for Epidemiologic Research (CDC WONDER) Database

**DOI:** 10.7759/cureus.68803

**Published:** 2024-09-06

**Authors:** Shamsul Arefin, Amruth A Alluri, Mousumi Barua, Tirth M Patel, Salini Krishnarao Kandhalu

**Affiliations:** 1 Department of Internal Medicine, Nottingham University Hospitals NHS Trust, Nottingham, GBR; 2 Department of Internal Medicine, American University of the Caribbean School of Medicine, Cupecoy, SXM; 3 School of Public Health and Health Professions, University at Buffalo, Buffalo, USA; 4 Department of Internal Medicine, Bukovinian State Medical University, Chernivtsi, UKR; 5 Department of Internal Medicine, Maharajah Institute of Medical Sciences, Vizianagaram, IND

**Keywords:** cardiac arrest, cdc wonder, disparities, mortality, retrospective, rural, trends, urban

## Abstract

Introduction

The United States includes diverse geographic areas with distinct urban and rural settings. Urban areas served with higher health services and the rural regions with restricted facilities. This disparity results in higher rural mortality rates. Thus, the study uses the Centers for Disease Control and Prevention Wide-Ranging Online Data for Epidemiologic Research (CDC WONDER) database to assess the disparities in cardiac arrest mortality rates in urban versus rural areas.

Methods

This is a retrospective study to assess trends in overall mortality rates for urban versus rural areas in the United States between 1999 and 2020, using the CDC WONDER data for cardiac arrest (ICD-10 CODE I46), extracted on May 25, 2024. Urban/rural classification was based on the Metropolitan 2013 scheme. Statistical analysis was done via RStudio v.4.3.3 and included measures of central tendency, mortality rates per 100,000, and plotting of temporal trends.

Results

Between 1999 and 2020, the total number of deaths due to cardiac arrest in rural and urban areas was 103,115 and 262,505, respectively. Among the age groups, infants <1 year and elderly >85 years showed a high mortality rate in rural areas compared to urban areas. Gender analysis revealed both males (3.3 per 100,000) and females (3.52 per 100,000) had a high rural mortality rate, compared to urban rates of 1.51 and 1.54 per 100,000, respectively. Racial analysis showed that American Indian or Alaska Native and Asian or Pacific Islander populations had higher mortality in rural areas, with rates of 1.1 and 1.81 per 100,000, respectively, compared to the urban rates of 0.34 and 0.8 per 100,000.

Conclusion

Trends in mortality rate showed a general decline over time but the gap between urban and rural mortality persists, highlighting the need for continued efforts in rural areas.

## Introduction

Cardiac arrest, characterized by the sudden loss of heart function, can occur in individuals regardless of pre-existing heart conditions. If immediate intervention is not administered, cardiac arrest frequently results in death. In the United States, over 436,000 deaths annually are attributed to cardiac arrest [1]. Nearly 90% of out-of-hospital cardiac arrests (OHCAs) are fatal. Data from 2021 reveals that only 9.1% of adults with OHCA who received emergency medical services survived hospital discharge [2]. A closer examination by geographical area shows that while mortality rates declined across all settings, rural areas experienced a significantly higher death rate (8.1 per 100,000) compared to large urban areas (3.5 per 100,000).

Studying the disparities in cardiac arrest mortality rates between urban and rural areas in the United States is crucial for identifying gaps in healthcare access and quality. This research can reveal how differences in emergency medical services and resources impact survival rates, guiding policymakers in the targeted allocation of resources and improvement of response systems. By pinpointing specific challenges faced by rural areas, the study can inform interventions such as enhancing local emergency response capabilities, increasing public awareness, and improving healthcare infrastructure. Addressing these disparities is vital for reducing health inequalities and ensuring equitable chances of survival for individuals experiencing cardiac arrest, regardless of their geographic location.

Aims and objectives

The aim of this study is to analyze and compare the mortality rates of cardiac arrest between urban and rural areas in the United States to identify disparities in survival outcomes. The study also aims to identify any significant associations between demographic characteristics, such as age, gender, and race, and the mortality rates. By investigating the factors contributing to these differences, the study seeks to provide insights into the variations in emergency medical services and healthcare access. Ultimately, the goal is to inform targeted interventions and policy changes that can improve cardiac arrest survival rates and reduce healthcare disparities across diverse geographic regions.

## Materials and methods

This retrospective original research study was conducted using the Centers for Disease Control and Prevention Wide-Ranging Online Data for Epidemiologic Research (CDC WONDER) database [3]. Data was extracted on May 30, 2024, from the webpage of the CDC (https://wonder.cdc.gov/ucd-icd10.html) for cardiac arrest. The CDC WONDER database has been previously used to study mortality in various disease conditions [4-6]. Informed consent and the institutional review board approval were not applicable as the CDC WONDER database includes only de-identified publicly available data [6]. This study uses a cross‐sectional study design examining all individuals who died of cardiac arrest from 1999 to 2020.

We primarily examined the trends in overall mortality rates for urban versus rural populations in the United States for the years 1999-2020. The ICD code for cardiac arrest "I46," based on the International Classification of Diseases, Tenth Revision, Clinical Modification (ICD‐10 CM), was used as the search term for extracting data from the CDC WONDER website [7]. The data search was performed separately for different categories such as year of death, gender, race, age group, and region. The extracted data for all categories were then combined in Microsoft Excel for statistical analyses. Urban/rural classification was based on the Metropolitan 2013 classification scheme [8]. The term urban meant metropolitan areas that represented four categories: (i) large central metropolitan, (ii) large fringe metropolitan, (iii) medium metropolitan, and (iv) small metropolitan. Similarly, the term rural meant non-metropolitan areas that contained micropolitan and non-core areas in different counties of the United States. Age groups were categorized as follows: <1, 1-4, 5-14, 15-24, 25-34, 35-44, 45-54, 55-64, 65-74, 75-84, and 85+ years. Gender was included as a dichotomous variable (male and female). Race was categorized into American Indian or Alaska Native, Asian or Pacific Islander, Black or African American, and White.

Mortality rates were computed as the proportion of the number of deaths in the total population within a category per 100,000 individuals. Statistical analysis was performed using the R package [9]. Binomial tests were used to compare the mortality rates between urban and rural populations within each of the categories of age group, gender, and race. The statistical plot or graph was created using ggplot2, which was built within the R package [10].

## Results

The data on the distribution of mortality by metropolitan and non-metropolitan areas reveals significant differences between urban and rural regions. In urban areas, there are a total of 262,505 deaths. Medium metropolitan areas report the highest number of mortalities with 82,540 deaths, accounting for 31.4% of the urban total. This is followed by large fringe metropolitan areas with 70,426 deaths (26.8%), large central metropolitan areas with 63,906 deaths (24.3%), and small metropolitan areas with 45,633 deaths (15.8%). In contrast, rural areas have a total of 103,115 deaths. Within these areas, micropolitan regions exhibit a higher number of deaths at 54,323, representing 52.7% of the rural total, while non-core regions account for 48,792 deaths, or 47.3%. This analysis highlights that medium metropolitan areas within urban regions and micropolitan areas within rural regions have the highest mortality numbers, emphasizing the notable disparities in mortality distribution between these specific urban and rural parts.

Aggregate data of 365,620 deaths from 1999 to 2020 was obtained for cardiac arrest from the CDC WONDER database (Table 1).

**Table 1:** The absolute number of reported mortalities in urban and rural areas due to cardiac arrest from 1999 to 2020 as per the 2013 urbanization classification.

Type	n (%)
Urban (metropolitan area)	262,505
Large central metropolitan	63,906 (24.3%)
Large fringe metropolitan	70,426 (26.8%)
Medium metropolitan	82,540 (31.4%)
Small metropolitan	45,633 (15.8%)
Rural (non-metropolitan area)	103,115
Micropolitan	54,323 (52.7%)
Non-core	48,792 (47.3%)

**Figure 1:**
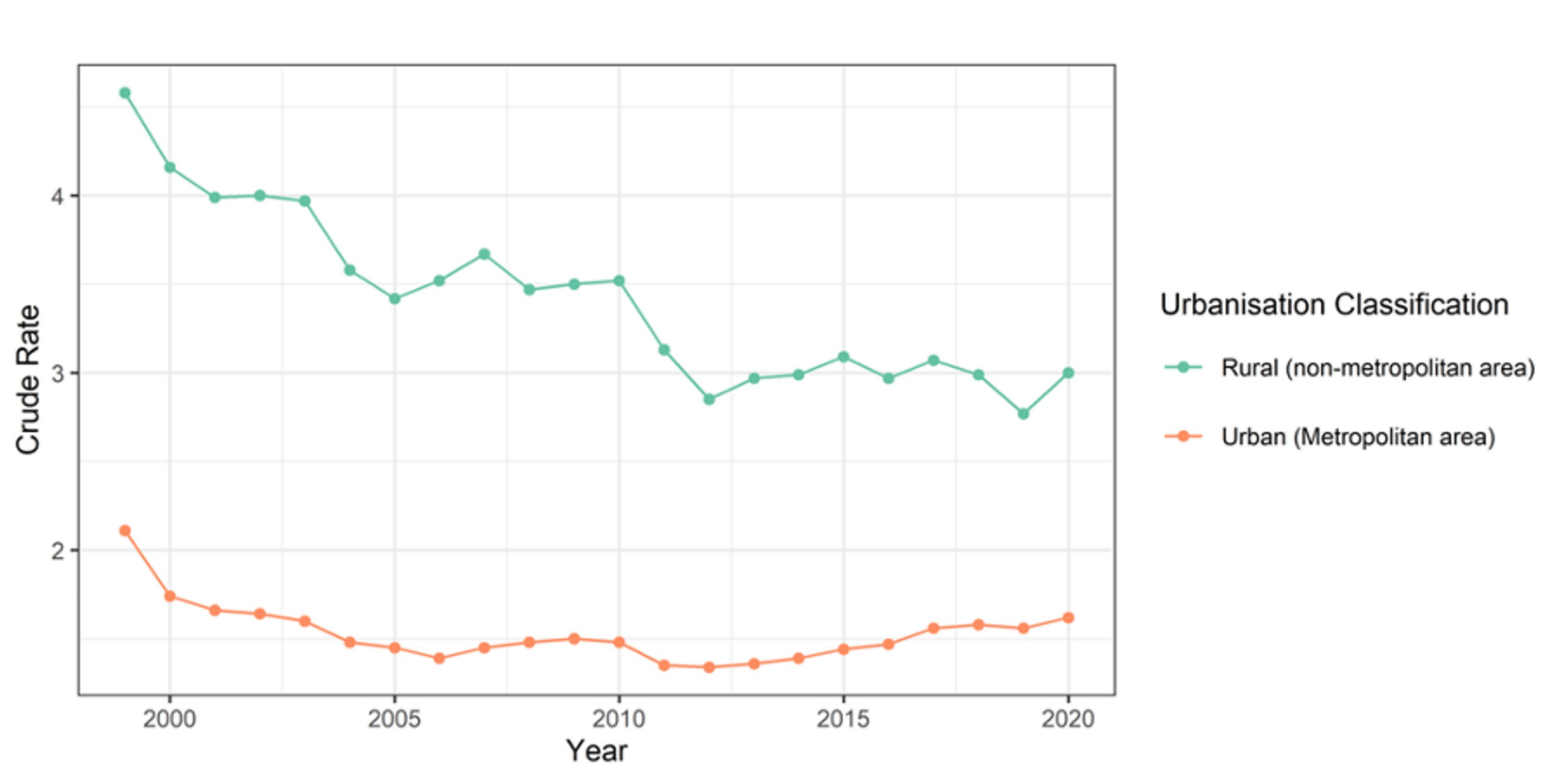
Crude mortality rates in urban and rural areas.

Figure 1 illustrates that mortality rates for cardiac arrest are significantly higher in rural areas across all years covered by the data. Despite this, rural mortality rates have shown a decrease from 1999 to 2012, after which they have remained stable. In contrast, urban mortality rates have been on a decline from 1999 to 2006. Between 2006 and 2012, the urban mortality rates largely plateaued, but starting from 2013, they have been steadily increasing.

The dataset reveals significant disparities in cardiac arrest mortality rates between urban and rural areas across different age groups, genders, and races, which have been depicted in Table 2. For infants (<1 year) and elderly adults (85+ years), rural areas exhibit notably higher mortality rates compared to urban areas. This trend is consistent among young adults (25-34 years) and older adults (65-74 years) as well. Gender analysis shows higher rural mortality for both females and males, with rates of 3.3 and 3.52 per 100,000, respectively, compared to urban rates of 1.51 and 1.54 per 100,000. Racial disparities indicate that American Indian or Alaska Native and Asian or Pacific Islander populations face higher mortality in rural areas, with rates of 1.1 and 1.81 per 100,000, respectively, compared to urban rates of 0.34 and 0.8 per 100,000.

**Table 2:** Mortality due to cardiac arrest in urban and rural areas based on age, gender, and race.

Variables	Urban			Rural			Binomial test
	Mortality	Total population	Mortality rate (per 100,000)	Mortality	Total population	Mortality rate (per 100,000)	P-value
Age groups							
<1 year	3784	2394185312	0.16	734	387051552	0.19	<0.001
1-4 years	1862	9585972416	0.02	307	1568264800	0.02	<0.001
5-14 years	1768	24644041056	0.01	292	4195063744	0.01	<0.001
15-24 years	3352	25592179680	0.01	673	4353219360	0.02	<0.001
25-34 years	7401	25697740000	0.03	1668	3745083488	0.04	<0.001
35-44 years	16611	25725321728	0.06	3604	4075832960	0.09	<0.001
45-54 years	37688	25235508448	0.15	8559	4446895936	0.19	<0.001
55-64 years	71471	20452379392	0.35	17394	4073167520	0.43	<0.001
65-74 years	126984	13375621664	0.95	32276	2959011936	1.09	<0.001
75-84 years	273442	7813886368	3.5	69601	1738244608	4	<0.001
85+ years	455209	3146384704	14.47	119620	678056960	17.64	<0.001
Gender							
Female	132076	8773260477	1.51	50008	1513737756	3.3	<0.001
Male	130417	8445166470	1.54	53107	1506877200	3.52	<0.001
Race							
American Indian or Alaska Native	633	188252325	0.34	848	76831248	1.1	<0.001
Asian or Pacific Islander	8624	1079920098	0.8	647	35820267	1.81	<0.001

## Discussion

Our retrospective research was conducted to study disparities in cardiac arrest mortality over 22 years. Data was collected from the CDC WONDER database. Our study revealed that mortality due to cardiac arrest was higher in urban areas than in rural areas in the United States, especially among certain demographic groups. This review offers a systemic look at the study of mortality trends for cardiac arrest in the United States. Over the last 22 years, mortality in urban areas has been increasing recently, while mortality in rural areas has decreased. However, the crude death rate in rural areas is significantly higher than in urban areas. When we stratified our results into age, gender, and race subgroups, the mortality rate was high among those older than 85 years, male gender, and Black or African American race in rural areas.

To improve healthcare policy and lower adverse occurrences, it is critical to comprehend the public health burden associated with cardiac arrest [11]. Significant advancements and novel technologies in cardiac arrest have been made in recent years, including improved understanding of post-resuscitation care, increased use of mechanical circulatory support (MCS), advancements in percutaneous coronary intervention (PCI), fresh research on therapeutic hypothermia, metabolic stabilization, and ventilator support [12]. Despite these advancements in healthcare, significant research has revealed substantial disparities in the treatment and results of cardiac arrest depending on factors such as age, sex, race, and area [13]. According to previous studies, after an OHCA, Black and Hispanic people are less likely than White people to receive bystander cardiopulmonary resuscitation (CPR) [14]. Black and Hispanic neighborhoods may not have as much access to dispatcher-assisted bystander CPR [15], and CPR training is less frequently provided there [16].

Regarding the mortalities caused by cardiac arrest, statistical data has been created, and the studies included in the meta-analysis show that there were 262,505 deaths in urban areas and 103,115 deaths in rural areas. It can be due to various underlying causes, such as a larger population in urban areas and more complicated cases in urban populations. In-hospital cardiac arrest incident rates are inversely correlated with hospital attributes such as small size, urban location, and a high percentage of Black patients [17].

However, our study showed crude death in rural areas has significantly higher mortality rates due to cardiac arrest in the last 22 years. High mortality rates in rural areas can be due to multiple factors. In a study conducted in Saudi Arabia, ambulances took 1.3 times longer to respond in rural areas compared to urban areas [18]. A study from Japan found that rural area patients with acute myocardial infarction were less likely to be directly transported to intervention-capable facilities, resulting in a longer time to primary intervention compared to metropolitan areas [19]. Another study showed that patients in the urban group had a 1.5 times higher chance of survival to discharge than those in the rural group, showing a significant difference [20].

Compared to rural communities, in urban areas, a more substantial number of potential bystanders present can efficiently initiate interventions such as calling emergency services and providing CPR during incidents of OHCA [20]. Also, it is commonly debated that the economic inequality in the United States has resulted in disparities in health outcomes [20]. All these factors can play a crucial role in high mortality rates in rural areas in the United States, which needs to be explored in further research to address the disparities between rural and urban mortality due to cardiac arrest.

Limitations

As demonstrated in various research studies, this epidemiologic study also has certain limitations. Firstly, our study includes statistical data from 1990 to 2020. The study did not include the data from 2021 to 2023, as the current data for these years was not available in the CDC WONDER database. In addition, we also observed a second limitation, as cardiac arrest was not further classified into different subcategories in the study. However, further research into different subclasses would be essential to advance the understanding of varying cardiac arrest-related death trends in the United States. The third limitation of our study was causes of death specific to cardiac arrest were not studied as data derived from the CDC WONDER database, and it does not list it. Similarly, socioeconomic causes and access to healthcare could not be studied.

## Conclusions

Disparities in urban versus rural mortality due to cardiac arrest in the United States have been significantly shown in our study, particularly in those older than 85 years, male gender, and Black or African American race in rural areas. It is crucial to comprehend the factors and trends contributing to cardiac arrest-related deaths thoroughly.

Future research should be undertaken to discover reasons for these disparities so that mortality related to cardiac arrest can be reduced. By recognizing the underlying factors contributing to cardiac arrest mortality, we can introduce effective interventions and policies to reduce the public health burden of cardiac arrest in the United States.
